# GPRED-GC: a Gene PREDiction model accounting for 5 ^′^- 3^′^ GC gradient

**DOI:** 10.1186/s12859-019-3047-3

**Published:** 2019-12-24

**Authors:** Prapaporn Techa-Angkoon, Kevin L. Childs, Yanni Sun

**Affiliations:** 1 Department of Electronic Engineering, City University of Hong Kong, Hong Kong SAR, China; 2 Department of Plant Biology, Michigan State University, East Lansing, 48824 MI USA; 3 Department of Computer Science and Engineering, Michigan State University, East Lansing, 48824 MI USA; 4 Department of Computer Science, Faculty of Science, Chiang Mai University, Chiang Mai, 50200 Thailand

**Keywords:** Gene finding, Plant genome gene prediction, Hidden Markov model, GC contents, Grass genomes

## Abstract

**Background:**

Gene is a key step in genome annotation. *Ab initio* gene prediction enables gene annotation of new genomes regardless of availability of homologous sequences. There exist a number of *ab initio* gene prediction tools and they have been widely used for gene annotation for various species. However, existing tools are not optimized for identifying genes with highly variable GC content. In addition, some genes in grass genomes exhibit a sharp 5 ^′^- 3^′^ decreasing GC content gradient, which is not carefully modeled by available gene prediction tools. Thus, there is still room to improve the sensitivity and accuracy for predicting genes with GC gradients.

**Results:**

In this work, we designed and implemented a new hidden Markov model (HMM)-based *ab initio* gene prediction tool, which is optimized for finding genes with highly variable GC contents, such as the genes with negative GC gradients in grass genomes. We tested the tool on three datasets from *Arabidopsis thaliana* and *Oryza sativa*. The results showed that our tool can identify genes missed by existing tools due to the highly variable GC contents.

**Conclusions:**

GPRED-GC can effectively predict genes with highly variable GC contents without manual intervention. It provides a useful complementary tool to existing ones such as Augustus for more sensitive gene discovery. The source code is freely available at https://sourceforge.net/projects/gpred-gc/.

## Background

Identification and annotation of genes in genomic sequences is a key step for functional analysis of a genome. The goal of gene annotation is to identify the location and structure of protein-coding genes in genomic sequences. Computational gene prediction methods can be broadly divided into two main categories: *ab initio* methods and homology-based methods. *Ab initio* gene prediction tools can predict genes in the query sequence without relying on the availability of homologs. A majority of *ab initio* gene prediction tools rely on hidden Markov models (HMMs), which describe different gene structural elements such as UTRs, exons, introns, etc. Given a sequence, we can use HMMs to infer the most probable path corresponding to an annotation of gene structure. Gene prediction tools such as GENSCAN [1, 2], GENEID [3], HMMGene [4], GeneMark.hmm [5], GlimmerHMM [6], FGENESH [7], SNAP [8], and AUGUSTUS [9] belong to the first category. The second category contains comparative gene prediction tools, which compare a query sequence with homologous sequences of related species and employ their sequence similarity for gene annotation. The examples of the second group include GENEWISE [10], GENOMESCAN [11], AGenDA [12, 13], TWINSCAN [14], SGP2 [15], DOUBLESCAN [16], CEM [17], SLAM [18], etc. There are also some machine learning based gene prediction programs [19–21], which are usually designed for prokaryotes such as metagenomic data rather than complicated gene structures containing introns.

Using more information such as homologous sequences has potential to produce better results. However, as a large number of new genomes are being sequenced using next-generation sequencing platforms, closely-related species are not always available. Thus, *ab initio* gene prediction tools play a significant role to find novel genes in the sequences without a priori known homologs. Note that some tools incorporate both HMMs and homologous sequences for boosting gene prediction performance. If a tool can conduct gene prediction without homologous sequences, it is classified into the first category.

### GC content-dependent gene prediction

As the base composition and the exon length distributions can differ significantly for genes with different GC contents, some gene prediction tools employ GC content-dependent training [1, 2, 9, 22, 23]. In animals, genome isochores are regions of the genome with different GC contents, and it has been shown that the GC content of animal genes closely matches the GC content of the isochore in which the gene is found [24]. The AUGUSTUS gene prediction program has a mode that creates independent HMMs based on the GC content of the genomic region that is being processed [9]. Both theoretical analysis and empirical results have shown that GC content-dependent training greatly improves the gene prediction accuracy and sensitivity.

In plants, isochores do not exist, and it has been shown that the GC content of plant genes is not correlated with the GC content of the genomic region in which the gene is found. Furthermore, in grasses such as *Oryza sativa* (rice), genes can be characterized as having either a high GC or low GC content whereas most non-grass species such as the model species *Arabidopsis thaliana* (thale cress) have genes with a narrow gene GC content distribution. Using a single HMM to predict these two classes of genes in *O. sativa* was shown to be less accurate than using a gene prediction protocol that was aware of the high and low GC genes in grasses. Bowman et al. [24] trained three HMM programs on low, medium and high GC genes. All HMMs were used to make gene predictions, but only the best prediction that was most congruent with available evidence was retained. This method improved gene predictions compared to a gene prediction protocol that was not GC aware.

**Fig. 1:**
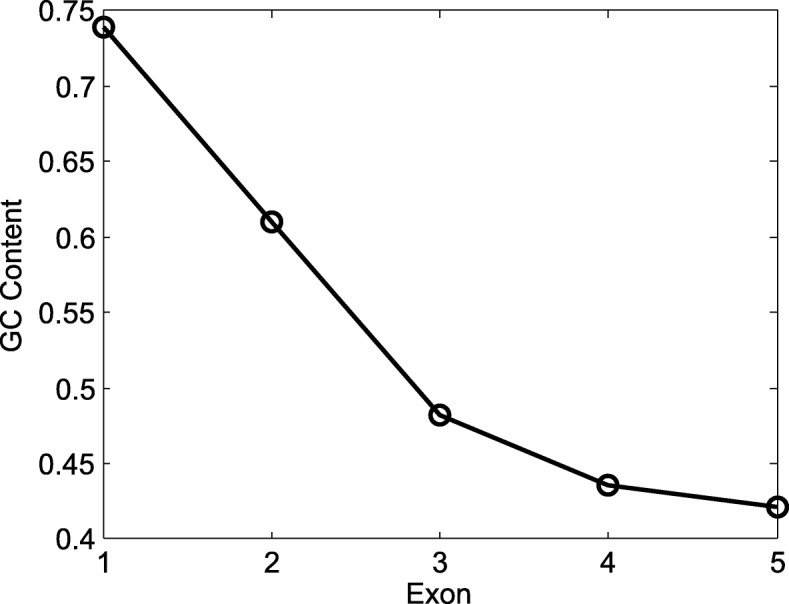
A gene LOC_Os03g44820.1 with GC content gradient from *Oryza sativa* data set. X-axis represents each exon inside the gene. Y-axis represents the GC content

While the method of Bowman et al. [24] is an improvement over other gene prediction programs, it is a heuristic that can be improved upon by an modification of the basic structure of the underlying gene prediction HMM. Furthermore, many grass genes exhibit a sharp 5 ^′^- 3^′^ decreasing GC content gradient [25], [26], which is not carefully modeled by existing gene prediction tools and Bowman’s method. As a result, these tools have unsatisfactory sensitivity and accuracy for predicting genes with GC gradients. Figure 1 illustrates an example of a gene with descending slope of GC content in *Oryza sativa* data set.

To address these limitations, we propose a new gene prediction model with two advantages: 1) our model can predict genes with GC gradient with higher sensitivity and accuracy without manual intervention; 2) our unified model is optimized for genes of variant GC content and 5 ^′^- 3^′^ changing patterns.

## Methods

**Fig. 2:**
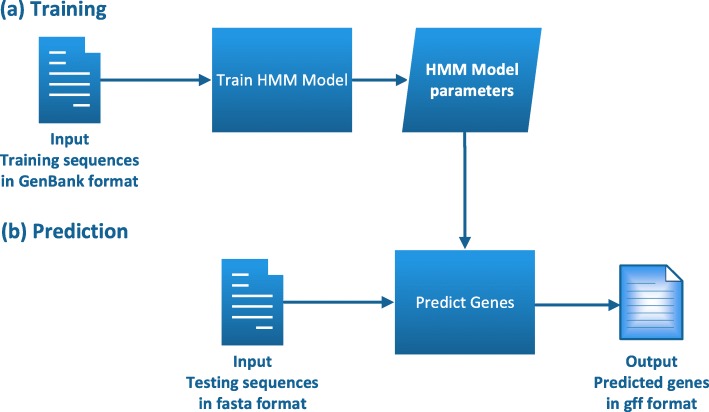
An overview of the training and predicting genes. (**a**) Training. (**b**) Prediction

In this section, we describe GPRED-GC, a tool that predicts genes with 5 ^′^- 3^′^ GC gradient. The flowchart for training and annotating genes is shown in Fig. 2. The main novelty of our method is a modified hidden Markov model (HMM) that distinguishes exons of different GC content. The HMM has a similar topology to the one used in AUGUSTUS [22, 23] and many other de novo gene prediction tools [1, 4]. The major difference is that our model is designed to handle various GC contents and 5 ^′^- 3^′^ changing patterns inside coding regions.

### The hidden Markov model of GPRED-GC

An HMM is a probabilistic sequence model with successful application for gene prediction. It models the key sequence features such as exons and introns in a gene and can be trained using annotated gene sets. Once the model is built, it can be applied to search for genes and annotate the gene structures using existing algorithms designed for HMMs, such as the Viterbi algorithm. Essentially, an optimal state path in an HMM that can maximize the likelihood or posterior probability of a query being produced by the model can be used to label each base in the query sequence.

**Fig. 3:**
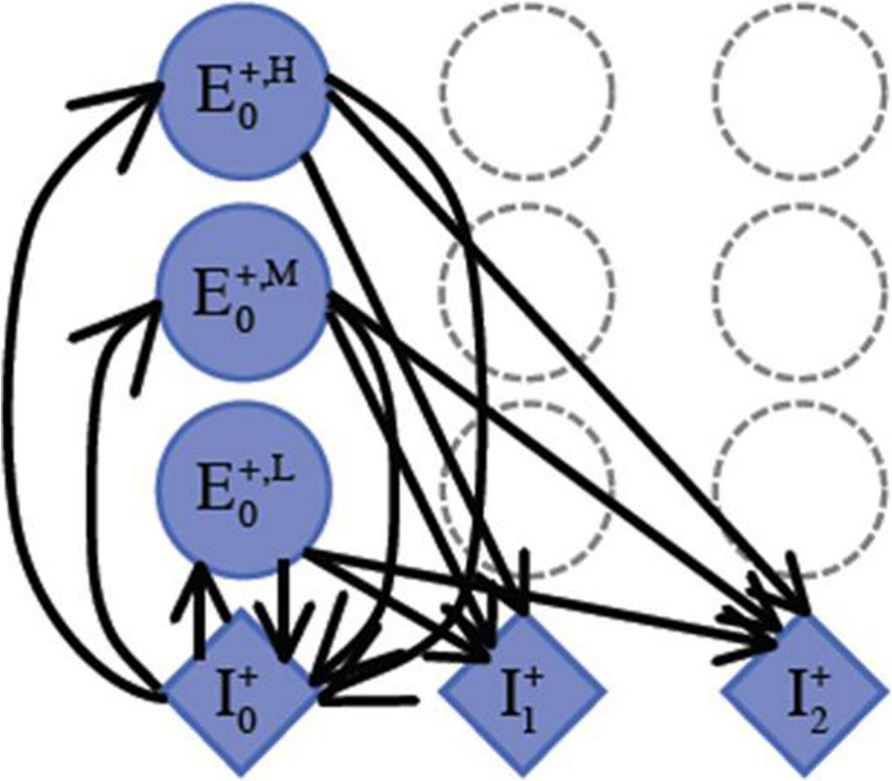
For each internal exon, three states ($E^{\text {+,H}}_{0}$, *E*0+,M,*E*0+,L) are used to model exons of high, medium, and low GC content. This figure only illustrates three internal exon states for one phase on the plus strand (corresponding to one reading frame). The internal exons of other phases, the initial exon, the terminal exon, and the single exon all have three states for high, medium, and low GC content. Genes of various GC contents and gradients can be represented as various paths through the exon states

As AUGUSTUS is a popular plant gene prediction tool, we use the generalized Hidden Markov Model from AUGUSTUS [22] as the base model. The essential difference is that instead of using one state to represent an exon, we have three states to model exons of high, medium, and low GC contents. Figure 3 illustrates the major difference for an exon state in a standard HMM and our HMM, which incorporates changes of GC contents across the genes.

Here, we make an assumption that the GC content change inside exons is relatively small. Although we can use a window-based model inside each exon to further refine the representation of GC gradient, it will significantly increase the model complexity. By only distinguishing exons of different GC contents, we have a better tradeoff between the model complexity and the model resolution.

For single exon genes, three states $\left (E^{\mathrm {H}}_{\text {single}}\right.$, $E^{\mathrm {M}}_{\text {single}}$, $\left.E^{\mathrm {L}}_{\text {single}}\right)$ are created. For the initial exon, three states $\left (E^{0}_{\text {init H}},\right.$$\left.E^{0}_{\text {init M}}\right.$, $\left.E^{0}_{\text {init L}}\right)$ are used to model exons of high, medium, and low GC content. Moreover, the initial exons of other phases, the internal exons of all phases, and the terminal exon all have three states for high, medium, and low GC content. Genes of variant GC changing patterns can be represented by the new exon states.

The added exon states allow the HMM to predict genes of various GC gradients with higher accuracy. For example, genes of negative GC gradient tend to be represented by a path starting with *E*^H^ and ending with *E*^L^. Genes with high GC content and moderate gradient tend to be produced by a path mainly consisting of *E*^H^.

### The states in the HMM

In total, the model of GPRED-GC has the following 79 states in set *Q*:

$$ \begin{aligned} & \{IR, E^{\mathrm{H}}_{\text{single}}, E^{\mathrm{M}}_{\text{single}}, E^{\mathrm{L}}_{\text{single}},E^{\mathrm{H}}_{\text{term}}, E^{\mathrm{M}}_{\text{term}}, E^{\mathrm{L}}_{\text{term}} \} \bigcup \\ & \{rE^{\mathrm{H}}_{\text{single}}, rE^{\mathrm{M}}_{\text{single}}, rE^{\mathrm{L}}_{\text{single}}, rE^{\mathrm{H}}_{\text{init}}, rE^{\mathrm{M}}_{\text{init}}, rE^{\mathrm{L}}_{\text{init}} \} \bigcup \\ & \bigcup_{i=0}^{2} \{E^{\mathrm{i}}_{\text{init H}}, E^{\mathrm{i}}_{\text{init M}}, E^{\mathrm{i}}_{\text{init L}}, DSS^{\mathrm{i}}, I^{\mathrm{i}}_{\text{short}}, I^{\mathrm{i}}_{\text{fixed}}, I^{\mathrm{i}}_{\text{geo}}\} \bigcup \\ \end{aligned}  $$

1 $$ \begin{aligned} & \bigcup_{i=0}^{2} \{ASS^{\mathrm{i}}, E^{\mathrm{i}}_{\mathrm{H}}, E^{\mathrm{i}}_{\mathrm{M}}, E^{\mathrm{i}}_{\mathrm{L}} \} \bigcup \\ & \bigcup_{i=0}^{2} \{rE^{\mathrm{i}}_{\text{term H}}, rE^{\mathrm{i}}_{\text{term M}}, rE^{\mathrm{i}}_{\text{term L}}, rDSS^{\mathrm{i}}, \} \bigcup \\ & \bigcup_{i=0}^{2} \{rI^{\mathrm{i}}_{\text{short}}, rI^{\mathrm{i}}_{\text{fixed}}, rI^{\mathrm{i}}_{\text{geo}}, rASS^{\mathrm{i}}, rE^{\mathrm{i}}_{\mathrm{H}}, rE^{\mathrm{i}}_{\mathrm{M}}, rE^{\mathrm{i}}_{\mathrm{L}} \} \\ \end{aligned}  $$

**Fig. 4:**
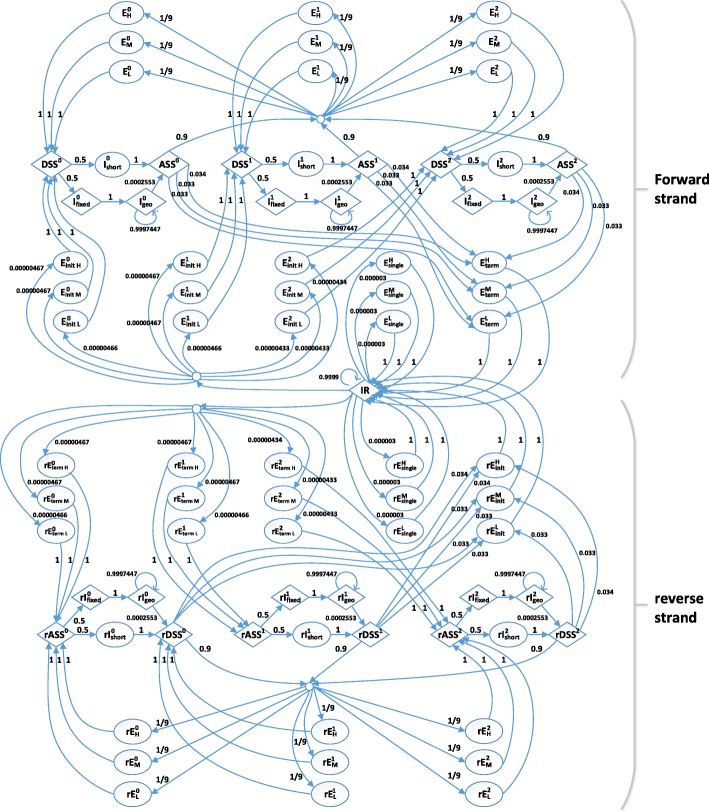
The state diagram of GPRED-GC. The states beginning with r represents the reverse strand. $E^{\mathrm {H}}_{\text {single}}$: a single exon of high GC content. $E^{\mathrm {M}}_{\text {single}}$: a single exon of medium GC content. $E^{\mathrm {L}}_{\text {single}}$: a single exon of low GC content. *E*_init H_: the initial coding exon of a multi-exon gene with high GC content. *E*_init M_ : the initial exon of a multi-exon gene with medium GC content. *E*_init L_: the initial exon of a multi-exon gene with low GC content. *DSS*: a donor splice site. *I*_short_: an intron emitting at most *d* nucleotides. *I*_fixed_: a longer intron with the first *d* nucleotides. *I*_geo_: a longer intron emitting one nucleotide at a time after the first *d* nucleotides. *ASS*: an acceptor splice site with branch point. *E*_H_: an internal coding exon of a multi-exon gene with high GC content. *E*_M_: the internal exon of a multi-exon gene with medium GC content. *E*_L_: the internal exon of a multi-exon gene with low GC content. $E^{\mathrm {H}}_{\text {term}}$: the last coding exon of a multi-exon gene with high GC content. $E^{\mathrm {M}}_{\text {term}}$: the terminal exon of a multi-exon gene with medium GC content. $E^{\mathrm {L}}_{\text {term}}$: the terminal exon of a multi-exon gene with low GC content. *IR*: intergenic region. Diamonds represent the states that emit fixed length strings. Ovals represent the states including explicit length distribution. The numbers at the arrows show the transition probabilities. The transition probabilities incident to new exon states are derived using equal divisions (strategy 1). The exponents 0, 1, and 2 represent the reading frame phase. For an exon state, this is the position of the last base of the exon in its codon. For the other states, the exponent are the preceding-exon phase. The small circles represent silent states

Figure 4 is a schematic representation of the HMM in our work. In the upper half of Fig. 4, the states represent protein-coding genes on the forward strand. The state *IR* stands for the intergenic region. In the lower half of Fig. 4, the states represent protein-coding genes on the reverse strand. Each state on the reverse strand begins with ‘r’. They have the consistent biological meaning with the states on the forward strand. The superscript on the reverse strand represents the reading frame phase of an exon. Thus, there are nine states for a terminal exon and three states for an initial exon considering high, medium, and low GC contents.

Similar to existing HMMs for gene prediction, our HMM is also a general HMM, which supports length distribution and the Markov model emission of the exons. For each exon state on both forward strand and reverse strand, the exon length distribution is computed on the corresponding exons, respectively. Similarly, different inhomogeneous kth-order Markov models (by default *k*=4) for each exon state are derived separately.

### New transitions in our HMM

With new states representing exons of different GC contents, new transitions incident to these new states are added. In this section, we describe how we compute the transition probabilities for the new edges.

In Fig. 4, the arrows represent the transitions between states in the state set *Q* with non-zero probabilities. The transitions from and to the intron states are the same as those of states described in the AUGUSTUS model [22]. For GPRED-GC, we consider two strategies for computing transition probabilities. **First strategy**, we use a very simple strategy by dividing the known transition probabilities concerning the exon states of AUGUSTUS equally for three exon states of high, medium, and low GC contents. This strategy can be used when we have very limited training data. Our hypothesis is that they start with equal probabilities. Figure 4 includes the transition probabilities of this strategy. **The second strategy** is a standard method based on maximum likelihood training. We compute the transition probabilities using the maximum likelihood estimation from the training data. In the following equation, *a*_*kl*_ is the transition probability for *k*,*l*∈*Q*. *A*_*kl*_ is the number of observed transitions from the state *k* to state *l* in training data. The maximum likelihood estimator is defined as

2 $$ a_{kl} = \frac{A_{kl}}{\sum_{q \in Q}A_{kq} }  $$

To avoid zero probabilities due to sparse/insufficient training data, we add pseudocounts to the observed frequencies to reflect prior biases regarding the probability values. Given pseudocounts *r*_*kl*_, we define $A^{\prime }_{kl}$ as

3 $$ A'_{kl} = A_{kl} + r_{kl}  $$

Usually, with the Laplace method, all *r*_*kl*_ equal to 1.

The performance comparison of the two strategies for computing transition probabilities will be shown in the Results and discussion Section.

## Results and discussion

To evaluate the performance of GPRED-GC, we tested GPRED-GC on three sets of data from *A. thaliana* and *O. sativa*. The first data set on *A. thaliana* was downloaded from the server of Augustus [27]. The other two were from *O. sativa*, obtained from the MSU Rice Annotation Project and from Stanke et al. [28], respectively. *A. thaliana* is a dicotyledenous plant and not a grass species. The genes in *A. thaliana* do not have GC-gradients that are common to genes from grasses. We expect that our program should achieve similar performance to other *ab initio* gene prediction programs on gene prediction for *A. thaliana*. For the data sets from *O. sativa*, we expect to observe improved performance of gene prediction. As our HMM is modified from the HMM in Augustus, we compared our results to the output of regular AUGUSTUS *ab initio* gene finding program. In particular, we focus on examining the performance of our method on identifying genes with sharp change of GC content.

### Evaluation metrics

We adopted the standard evaluation metrics [22] for gene prediction: sensitivity and specificity. The sensitivity and specificity are computed at three levels: the nucleotide level, the exon level, and the gene level. The sensitivity and the specificity are defined as

4 $$ Sensitivity (Sen) = \frac{TP}{TP+FN}  $$

5 $$ Specificity (Spe) = \frac{TP}{TP+FP}  $$

*TP* (true positive) represents the number of correctly predicted features (coding nucleotides, exons, or genes). *FN* (false negative) represents the number of annotated features that are not correctly predicted by a gene annotation program. *FP* represents the number of predicted features that are not annotated. At each level, we report both the sensitivity and specificity. Sensitivity is the percentage of correctly predicted features in the set of all annotated features. Specificity is the percentage of correctly predicted features in the set of all predicted features. Specificity is also called positive predictive value (PPV) in other literature. At the exon level, a predicted exon will be correct if both splice sites are identical to their labeled positions. At the gene level, a predicted gene is considered correct if all exons are correctly identified, and no additional exons are identified in the gene. The predicted partial genes are evaluated similarly. The forward and reverse strands are considered as different sequences.

### Gene prediction on *A. thaliana*

We trained our HMM for *A. thaliana* using the training set from Stanke’s website [27]. The data set contained 249 genomic regions. There were two single exon genes and 247 multi-exon genes in the data set.

#### Training the HMM model

To train our HMM, we calculated the GC contents for all of the exons and classified them as high, medium, and low using specified cutoffs. For GPRED-GC, we have two cutoffs: lowT and highT. If an exon has GC content below lowT, it is classified as low GC content. If an exon has GC content above highT, it is labeled as high GC content. Otherwise, it is labeled as medium. The detailed classification is summarized in Procedure 1.

**Fig. 5:**
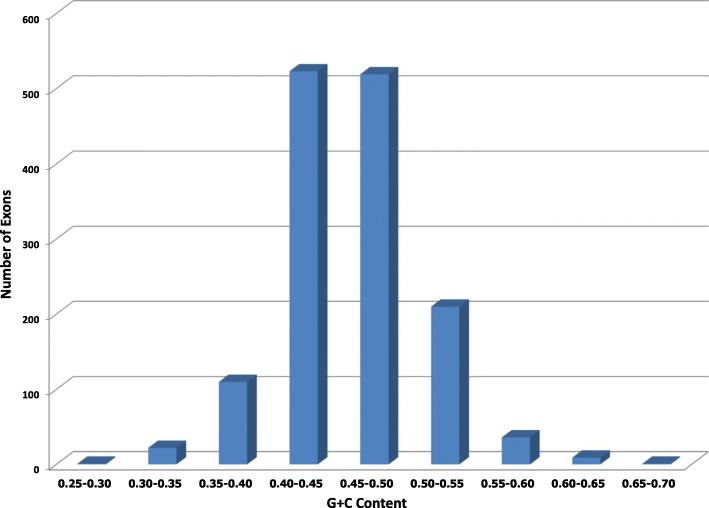
GC Content of exons in the *A. thaliana* data set


Figure 5 illustrates the distribution of exons by their GC contents for 1,431 exons in *A. thaliana* data set. Compared to the GC content distribution for exons in *O. sativa* (see the figure in Section Gene Prediction in *O. sativa*), the variation of GC contents of exons in *A. thaliana* is smaller.

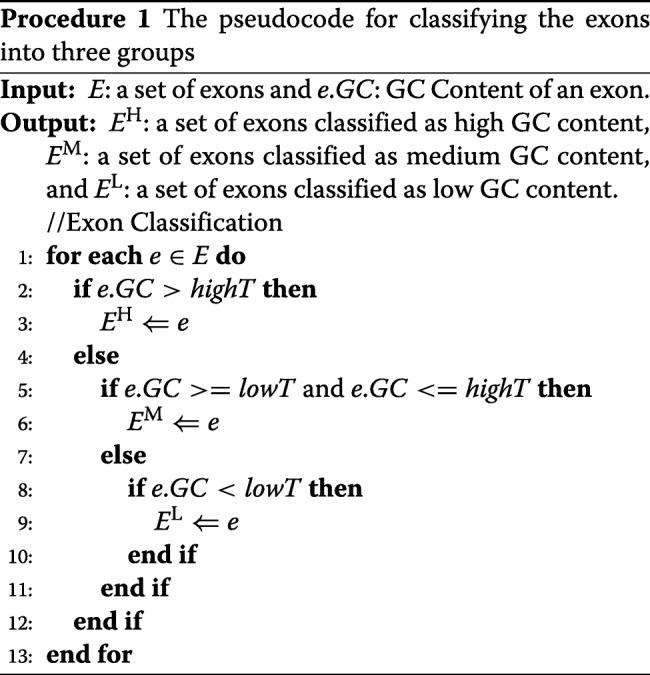


Table 1 shows the values of lowT and highT used in the experiments. Exons in the training set are classified into three groups based on lowT and highT cutoffs. Parameters are derived separately for different exon states.

**Table 1:** Performance comparison of gene prediction tools on *A. thaliana* with the transition probabilities divided into three equal portions

Program		AUGUSTUS	GPRED-GC			
			lowT=0.47	lowT=0.30	lowT=0.30	lowT=0.60
			highT=0.63	highT=0.60	highT=0.70	highT=0.70
Base	Sen	0.968	0.962	0.963	0.962	0.963
level	Spe	0.708	**0.709**	**0.710**	**0.710**	**0.710**
Exon	Sen	0.870	0.848	0.848	0.845	0.848
level	Spe	0.666	**0.669**	**0.679**	**0.680**	**0.679**
Gene	Sen	0.554	**0.565**	0.548	0.548	0.548
level	Spe	0.352	**0.360**	**0.354**	**0.354**	**0.354**
Time(Sec.)		40.3	52.4	52.8	54.2	53.0

**Bold number** indicates that sensitivity or specificity of GPRED-GC are higher than those of AUGUSTUS. Time (Sec.) is the running time of AUGUSTUS and GPRED-GC under different sets of thresholds on *A. thaliana* dataset in seconds. *Note:* The running time is the total running time of prediction

For all newly added exons of types *E*^*H*^, *E*^*L*^, and *E*^*M*^, their exon length distributions are computed. In addition, we calculated *k*th-order Markov Model (by default *k*=4) for each new exon state.

For computing transition probabilities, we used two strategies. First, we equally divided the probabilities of AUGUSTUS for three states of high, medium, and low GC contents. Second, we used maximum likelihood estimation to calculate transition probabilities.

We used 10-fold cross-validation for model training. It divided the training data set randomly into 10 subsets. The evaluation method is repeated 10 times. For each round, one of 10 subsets is designated as the test set and the other 9 subsets are put together for training. Then the average prediction accuracy of all 10 trials is calculated. The parameters maximizing the average prediction performance are kept as the default parameters.

#### Performance comparison between different gene prediction tools

We tested AUGUSTUS and GPRED-GC on the testing data set of *A. thaliana* [27], which has no overlap with the training data set. There were 74 genomic regions with 168 genes on the forward and reverse strand. Our program was modified from AUGUSTUS version 2.4 downloaded from [29]. Both original AUGUSTUS and GPRED-GC were tested using default input parameters.

Table 1 shows the comparison of the accuracy of AUGUSTUS and GPRED-GC with different thresholds of GC contents. In this experiment, the transition probabilities from intron to exons of different GC contents were equally divided into three portions. These experimental results show that GPRED-GC achieved slightly better sensitivity and specificity for gene level predictions. For base level and exon level predictions, GPRED-GC has higher specificity than AUGUSTUS. Overall, the performances of these two tools are comparable on this data set, which is expected for a non-grass genome that lack genes with widely varying GC contents or genes with negative GC gradients. In addition, the performance of GPRED-GC does not vary significantly with the change of the GC content cutoffs, mainly because the GC contents of the exons in this data set are clustered between 0.35 and 0.6.

We also implemented GPRED-GC by computing the transition probabilities from intron to different exon states using maximum likelihood estimation. Table 2 presents the accuracy comparison of AUGUSTUS and GPRED-GC with different cutoffs and trained transition probabilities. Using maximum likelihood estimation for computing the transition probabilities gave the better overall performance.

**Table 2:** Performance comparison of gene prediction tools on *A. thaliana* with the transition probabilities trained by computing maximum likelihood estimation

Program		AUGUSTUS	GPRED-GC			
			lowT=0.47	lowT=0.30	lowT=0.30	lowT=0.60
			highT=0.63	highT=0.60	highT=0.70	highT=0.70
Base	Sen	0.968	0.960	0.972	**0.972**	**0.972**
level	Spe	0.708	**0.711**	**0.709**	**0.709**	**0.709**
Exon	Sen	0.870	0.851	**0.882**	**0.882**	**0.882**
level	Spe	0.666	**0.674**	**0.677**	**0.677**	0.677
Gene	Sen	0.554	**0.560**	**0.565**	**0.565**	**0.565**
level	Spe	0.352	0.346	0.351	0.351	0.351
Time(Sec.)		40.3	51.1	57.7	56.4	57.7

The two tools have comparable performance. Time (Sec.) is the running time of AUGUSTUS and GPRED-GC under different sets of thresholds on *A. thaliana* dataset in seconds. *Note:* The running time represents the total running time of prediction

#### The uniquely predicted genes by GPRED-GC

As the major goal of GPRED-GC is to detect genes with highly variable GC contents, we evaluated this goal by examining the GC contents of uniquely identified genes by GPRED-GC. There were 14 uniquely identified genes by our tool and 149 shared genes. For all these genes, we computed their GC contents and the standard deviation (*SD*). In addition, we introduce another metric named “*GC-distance*", which is the largest difference of GC contents between all exons inside a gene. Thus, a gene with highly variable GC contents are more likely to have a big *SD* and also a large GC-distance.

**Fig. 6:**
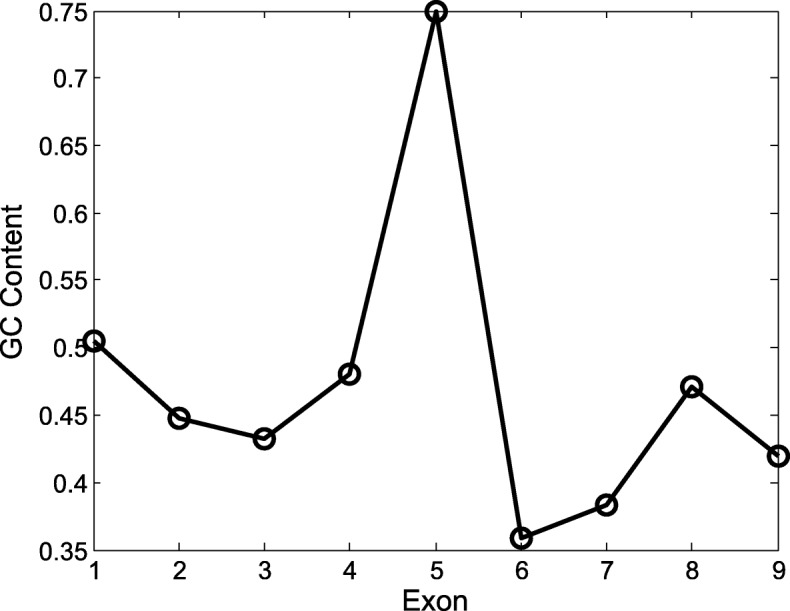
The GC content change across all exons in a predicted multi-exon gene of *A. thaliana*. This gene SEQ16AC003000G7G8 was predicted by GPRED-GC. X-axis represents the exon index. Y-axis represents the GC content

The experimental results demonstrated that the average *SD* of the uniquely predicted genes was 0.046. However, the average *SD* of the common ones was 0.033 which is smaller than the uniquely predicted genes. Also, the average GC-distance for uniquely found genes was 0.111. The average GC-distance of the common genes was only 0.087. As an example, a uniquely identified gene is reported in Fig. 6.

#### Running time analysis

The theoretical time complexity is *O*(|*Q*|*L*) where *Q* is the set of the states in the HMM and *L* is the query length. The actual running time is in Tables 1 and 2. As the total number of states in GPRED-GC is less than twice of the states in AUGUSTUS, the running time of GPRED-GC is comparable to AUGUSTUS.

### Gene prediction in *O. sativa*

**Fig. 7:**
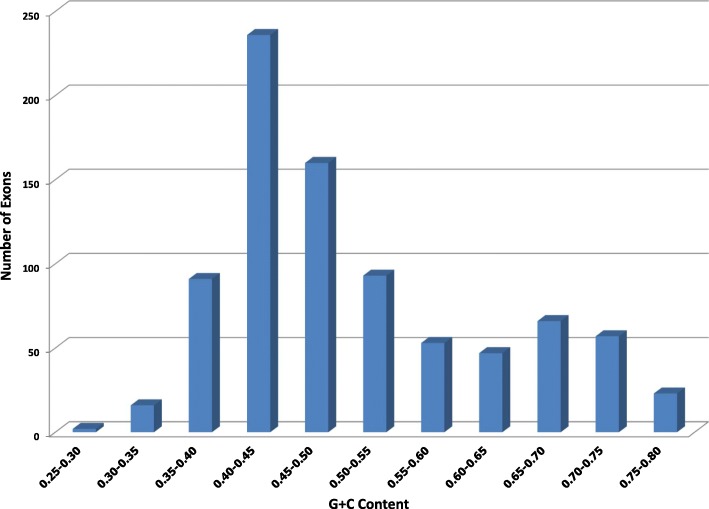
GC Content of the exons in the first data set of *O. sativa*

**Fig. 8:**
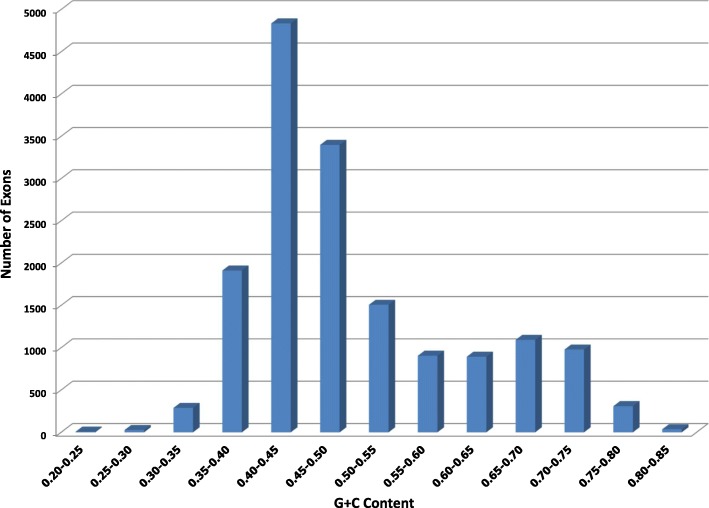
GC Content of the exons in the second *O. sativa* data set

We conducted two experiments using two different *O. sativa* data sets. The first *O. sativa* data set is part of the MSU Rice Genome Annotation Project [30], [31]. The second *O. sativa* data set was obtained from Stanke et al. [28]. Unlike *A. thaliana*, which has a set of gene predictions with high confidence, *O. sativa* does not have confidence descriptions assigned to gene predictions. Therefore, we choose two data sets on *O. sativa* to avoid the possible inaccurate annotations. These two data sets were constructed from different means and contain different sequences. The first data set is smaller than the second data set. Figure 7 presents the distribution of exons by their GC contents for 844 exons in the first *O. sativa* data set. Figure 8 shows the distribution of exons by their GC contents for 16,199 exons in the second *O. sativa* data set. According to Figs. 7 and 8, the GC content of the exons has a larger variation than that of *A. thaliana*. Thus, the main purpose of the experiments is to test whether our model can capture the change of GC content inside the genes.

#### Gene identification in the first *O. sativa* data set

The training data set consisted of 150 genomic regions with 11 single-exon genes and 139 multi-exon genes on forward strand and reverse strand. Again, we used 10-fold cross validation strategy for model training. The test data set contains 150 genomic regions with 13 single exon genes and 137 multi-exon genes on forward and reverse strands.

We compared GPRED-GC with AUGUSTUS in Table 3. In this experiment, we observed GC content change for exons inside each gene. For example, some genes tend to start with exons of high GC content and end with exons of low GC content. As the result, GPRED-GC achieved higher sensitivity than AUGUSTUS. GPRED-GC can improve both the sensitivity and the specificity at all levels using a cutoff of low GC content(0.40) and the cutoff of high GC content(0.60). With transition probabilities derived using maximum likelihood, the results are shown in Table 4.

**Table 3:** Performance comparison of gene prediction on the first *O. sativa* data set with the transition probabilities divided into three equal parts

Program		AUGUSTUS	GPRED-GC			
			lowT=0.39	lowT=0.35	lowT=0.50	lowT=0.40
			highT=0.61	highT=0.61	highT=0.60	highT=0.60
Base	Sen	0.839	**0.840**	0.831	**0.921**	**0.841**
level	Spe	0.892	**0.902**	**0.898**	0.883	**0.901**
Exon	Sen	0.613	**0.617**	0.589	**0.698**	**0.617**
level	Spe	0.694	**0.733**	**0.715**	0.692	**0.725**
Gene	Sen	0.260	**0.280**	**0.267**	**0.267**	**0.287**
level	Spe	0.235	**0.261**	**0.250**	0.234	**0.267**
Time(Sec.)		37.6	58.1	58.2	57.0	56.0

**Bold number** indicates that sensitivity or specificity of GPRED-GC are higher than those of AUGUSTUS. Time (Sec.) is the running time of AUGUSTUS and GPRED-GC under different sets of thresholds on the first *O. sativa* dataset in seconds. *Note:* The running time is the total running time of prediction

**Table 4:** Performance comparison of gene prediction on the first *O. sativa* data set with the transition probabilities trained using maximum likelihood estimation

Program		AUGUSTUS	GPRED-GC			
			lowT=0.39	lowT=0.35	lowT=0.50	lowT=0.40
			highT=0.61	highT=0.61	highT=0.60	highT=0.60
Base	Sen	0.839	**0.850**	**0.847**	**0.923**	**0.847**
level	Spe	0.892	**0.898**	**0.898**	0.876	**0.899**
Exon	Sen	0.613	**0.633**	**0.62**	**0.707**	**0.629**
level	Spe	0.694	**0.732**	**0.716**	0.670	**0.727**
Gene	Sen	0.260	0.253	0.253	**0.267**	**0.267**
level	Spe	0.235	**0.235**	**0.235**	0.227	**0.247**
Time(Sec.)		37.6	57.4	57.8	56.0	57.4

**Bold number** indicates that sensitivity or specificity of GPRED-GC are higher than those of AUGUSTUS. Time (Sec.) is the running time of AUGUSTUS and GPRED-GC under different sets of thresholds on the first *O. sativa* dataset in seconds. *Note:* The running time shows the total running time of prediction

Furthermore, we compared changes in GC contents of the uniquely identified genes by GPRED-GC and the common genes shared by AUGUSTUS and GPRED-GC. There were four uniquely predicted genes by GPRED-GC and 143 common genes. We compared the uniquely identified genes and common ones in terms of *SD* and GC-distance for each protein-coding gene. The results showed that the average *SD* of the uniquely predicted genes (0.098) was higher than that of common genes (0.075). Also, the average GC-distance of the uniquely found genes by GPRED-GC (0.241) was bigger than that of common genes (0.171).

**Fig. 9:**
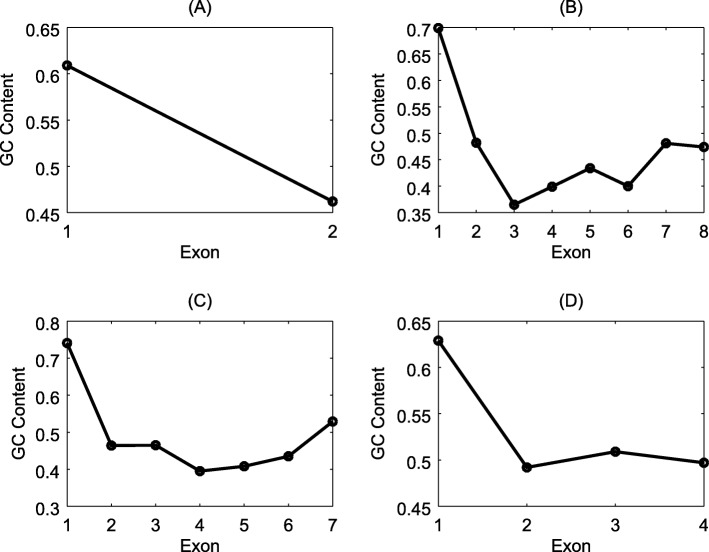
Genes of the first *O. sativa* data set predicted correctly by GPRED-GC but missed or incorrectly annotated by Augustus. Four genes are listed in the four subplots: (**a**), (**b**), (**c**), and (**d**). X-axis represents the exon index inside a gene. Y-axis represents GC content

Figure 9 plots the GC contents of the genes that can be correctly predicted by GPRED-GC but miss-annotated by regular AUGUSTUS. All these genes have a negative GC gradient.

#### Running time on the first *O. sativa* data set

The running times of AUGUSTUS and GPRED-GC with different sets of thresholds are compared in Table 3 and Table 4.

#### Finding genes in the second *O. sativa* data set

The second *O. sativa* data set was provided by Stanke et al. [28]. The detailed information about these genes can be found in the authors’ paper. Here we provide a brief summary about the genes in this data set. First, Stanke et al. made a genbank file from the genome and the gff file. Second, they constructed a set with both 5’ and 3’ UTRs annotated. Then, they selected genes with both UTRs and CDSes (from a visual inspection in JBrowse against panicle and leaf RNA-Seq STAR alignments). Fourth, they identified genes with errors only and removed the sequences with errors. Finally, the data set contains 1000 genes, which consist of 128 manually selected and 872 randomly chosen ones (from among filtered genes with both UTRs annotated). The HMM for *O. sativa* was trained on a selection of 800 genes with UTRs from phytozome [32]. For the training data set, there were 187 single exon genes and 613 multi-exon genes on the forward and reverse strands.

To assess the performance, we used the remaining 200 genes as the test set to avoid any overlap with the training set. The testing data set consisted of 40 single-exon genes and 160 multi-exon genes on the forward and reverse strands. Tables 5 and 6 compare the accuracy of AUGUSTUS and GPRED-GC on the test set. Table 5 shows the results of the prediction using the equal transition probabilities while Table 6 contains the prediction results of the HMM whose transition probabilities were trained using maximum likelihood. Both models shows improved accuracy compared to AUGUSTUS.

**Table 5:** Performance comparison of gene prediction tools on the second *O. sativa* data set with the transition probabilities divided into three equal parts

Program		AUGUSTUS	GPRED-GC			
			lowT=0.31	lowT=0.49	lowT=0.30	lowT=0.60
			highT=0.52	highT=0.52	highT=0.50	highT=0.70
Base	Sen	0.859	**0.942**	**0.950**	**0.937**	0.840
level	Spe	0.619	0.607	0.597	0.590	**0.622**
Exon	Sen	0.670	**0.768**	**0.781**	**0.748**	0.630
level	Spe	0.552	0.546	**0.553**	0.520	**0.559**
Gene	Sen	0.355	**0.400**	**0.400**	**0.355**	**0.365**
level	Spe	0.191	**0.211**	**0.205**	0.177	**0.204**
Time(Sec.)		48.2	60.2	60.8	60.8	59.0

The transition probabilities were divided into three equal parts. **Bold number** indicates that sensitivity or specificity of GPRED-GC are higher than those of AUGUSTUS. Time (Sec.) is the running time of AUGUSTUS and GPRED-GC under different sets of thresholds on the second *O. sativa* dataset in seconds. *Note:* The total running time of prediction is presented

**Table 6:** Performance comparison of gene prediction tools on the second *O. sativa* data set with the transition probabilities trained using maximum likelihood estimation

Program		AUGUSTUS	GPRED-GC			
			lowT=0.31	lowT=0.49	lowT=0.30	lowT=0.60
			highT=0.52	highT=0.52	highT=0.50	highT=0.70
Base	Sen	0.859	**0.955**	**0.945**	**0.948**	0.858
level	Spe	0.619	0.607	0.601	0.586	**0.620**
Exon	Sen	0.670	**0.798**	**0.769**	**0.765**	0.665
level	Spe	0.552	**0.565**	0.544	**0.572**	0.547
Gene	Sen	0.355	**0.425**	**0.360**	0.350	**0.370**
level	Spe	0.191	**0.217**	0.186	**0.170**	**0.204**
Time(Sec.)		48.2	64.9	62.4	63.5	60.3

**Bold number** indicates that sensitivity or specificity of GPRED-GC are higher than those of AUGUSTUS. Time (Sec.) is the running time of AUGUSTUS and GPRED-GC under different sets of thresholds on the second *O. sativa* dataset in seconds. *Note:* The running time is the total running time of prediction

By applying GPRED-GC of equal transition probabilities (strategy 1), the sensitivity of GPRED-GC at base level was enhanced from 0.859 to 0.942 for 0.31 lowT cutoff and 0.52 highT cutoff. At the exon level, the sensitivity of GPRED-GC was improved from 0.67 to 0.768. The specificity of AUGUSTUS is slightly better than that of GPRED-GC for the same cutoffs at the base level. At the gene level, GPRED-GC had better sensitivity and specificity (0.4 and 0.211, respectively). By using the transition probabilities trained via maximum likelihood, we observed a bigger improvement in the performance (see Table 6).

We conducted additional analysis using the results of lowT=0.31 and highT=0.52. The analysis confirms that GPRED-GC can detect the genes containing a GC content gradient. There were 26 uniquely predicted genes by GPRED-GC and 163 common genes shared by AUGUSTUS and GPRED-GC. Uniquely predicted genes had higher *SD* (0.099) than common genes (0.080). Besides, the average GC-distance (i.e. the difference between the exon with the highest GC content and the exon with the lowest GC content) for each protein-coding gene of the uniquely found genes are larger than that of the common genes (0.211 and 0.171, respectively). GPRED-GC miss-annotated 11 genes, which are correctly predicted by AUGUSTUS. For all these genes, we correctly predicted the initial and terminal exons but some of the internal exons’ starting and ending positions are not correctly computed. Overall, we correctly identified 26 more genes than AUGUSTUS while we missed 11 genes. There are 533 exons in all of these 37 genes. At the exon level for these genes, the sensitivities of GPRED-GC vs. AUGUSTUS are 0.838 and 0.627, respectively. The specificities of GPRED-GC VS AUGUSTUS are 0.639 and 0.543, respectively.

**Fig. 10:**
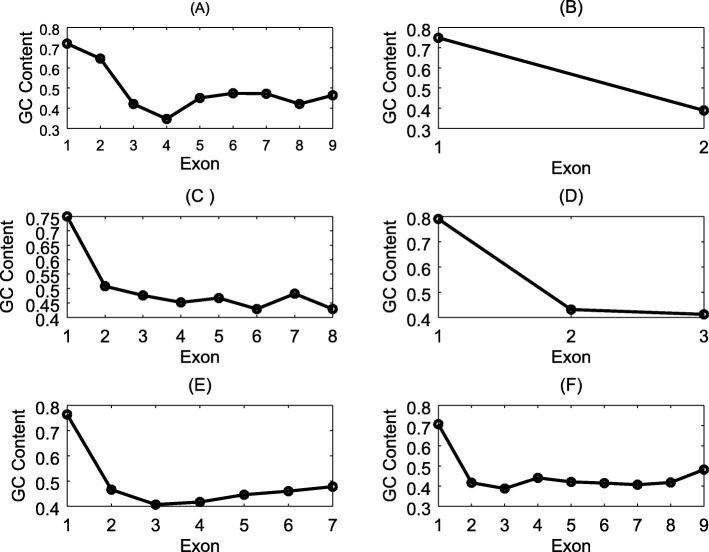
Summary of GC content profile of six genes correctly predicted by GPRED-GC. The names of the genes in each subplot are (**a**) LOC_Os03g44820.1, (**b**) LOC_Os04g52180.1, (**c**) LOC_Os04g52710.1, (**d**) LOC_Os05g30860.1, (**e**) LOC_Os06g11040.1, (**f**) LOC_Os10g03830.1, respectively from the second *O. sativa* data set. These genes cannot be detected or annotated correctly by AUGUSTUS. X-axis represents exon index inside the gene. Y-axis represents the GC content

Figure 10 illustrates the examples of genes correctly predicted by GPRED-GC but missed by AUGUSTUS. This is strong evidence showing that our tool can predict genes with changing GC contents.

#### How to determine the lowT and highT cutoffs?

Our experimental results have shown that the gene prediction performance is affected by the values of lowT and highT. In Table 6, setting lowT and highT to 0.31 and 0.52 achieved significantly better performance than 0.30 and 0.50. As such a small change can lead to a big difference, we investigated the reasons. Essentially, changing lowT and highT mainly changes the labels of exons of the training data. The numbers of exons classified as having high, medium, and low GC content may change. Meanwhile, the number of transitions involving these states may change too. Thus, we compared the corresponding parameters in the two HMMs for these two sets of cutoffs. While many parameters are identical, there are several differences as shown in Table 7.

**Table 7:** The comparison of the corresponding parameters in the two HMMs for these two sets of cutoffs

From	To	lowT=0.30, highT=0.50		lowT=0.31, highT=0.52	
		Transition probabilities	Training count	Transition probabilities	Training count
*ASS*^0^	$E^{1}_{\mathrm {H}}$	0.051961	212	0.045098	184
*ASS*^0^	$E^{1}_{\mathrm {M}}$	0.125490	510	0.132353	540
*ASS*^0^	$E^{1}_{\mathrm {L}}$	0.000980	4	0.000980	4
*ASS*^0^	$E^{2}_{\mathrm {H}}$	0.034314	140	0.027450	112
*ASS*^0^	$E^{2}_{\mathrm {M}}$	0.124510	508	0.129412	528
*ASS*^0^	$E^{2}_{\mathrm {L}}$	0.000980	4	0.002941	12
*ASS*^1^	$E^{0}_{\mathrm {H}}$	0.119469	216	0.101770	184
*ASS*^1^	$E^{0}_{\mathrm {M}}$	0.316372	572	0.334071	604
*ASS*^1^	$E^{0}_{\mathrm {L}}$	0.004425	8	0.004425	8
*ASS*^1^	$E^{\mathrm {H}}_{\text {term}}$	0.066372	120	0.055310	100
*ASS*^1^	$E^{\mathrm {M}}_{\text {term}}$	0.130531	236	0.139381	252
*ASS*^1^	$E^{\mathrm {L}}_{\text {term}}$	0.002212	4	0.004425	8
*rDSS*^0^	$rE^{2}_{\mathrm {H}}$	0.107246	148	0.092754	128
*rDSS*^0^	$rE^{2}_{\mathrm {M}}$	0.272464	376	0.284058	392
*rDSS*^0^	$rE^{2}_{\mathrm {L}}$	0	0	0.002899	4
*rDSS*^1^	$rE^{2}_{\mathrm {H}}$	0.074074	96	0.067901	88
*rDSS*^1^	$rE^{2}_{\mathrm {M}}$	0.379630	492	0.385802	500
*rDSS*^1^	$rE^{2}_{\mathrm {L}}$	0.003086	4	0.003086	4
*rDSS*^2^	$rE^{2}_{\mathrm {H}}$	0.099088	348	0.077449	272
*rDSS*^2^	$rE^{2}_{\mathrm {M}}$	0.407745	1432	0.428246	1504
*rDSS*^2^	$rE^{2}_{\mathrm {L}}$	0.001139	4	0.002278	8

Set1: lowT and highT are 0.30 and 0.50. Set2: lowT and highT are 0.31 and 0.52. The different probabilities before using pseudocount and their corresponding training counts are listed

For the cutoff set (0.30, 0.50), there are several edges that have zero or a very small number of training samples passing those edges. If the training case contains 0 samples for one edge, only the pseudocount will be used, leading to a very small transition probability. When the testing data has those cases, the overall generation probabilities tend to be small. In order to avoid any bias of training, our guidance is to choose the cutoffs so that the training data can cover all the edges/transitions.

#### Running time on the second *O. sativa* data set

The running times of AUGUSTUS and GPRED-GC with different sets of thresholds are compared in Tables 5 and 6. The two tools have comparable total running time.

## Conclusion

In this work, we provided an implementation of a HMM that is optimized for predicting protein-coding regions that have various GC content and 5 ^′^- 3^′^ changing patterns. Our experimental results showed that our program can identify genes that are missed by AUGUSTUS.

According to the previous studies, several directions can be improved. For gene prediction, some existing gene prediction tools can identify 5 ^′^UTR and 3 ^′^UTR regions. Currently, GPRED-GC is not able to identify UTR regions. We plan to extend GPRED-GC to accurately predict 5’UTR and 3’UTR regions. Existing gene prediction tools demonstrated that using extrinsic evidence derived from matches to an EST or protein database can improve the accuracy of gene prediction. We will further improve GPRED-GC accuracy by using hints from external sources if the data is available.

## Data Availability

GPRED-GC can be download from https://sourceforge.net/projects/gpred-gc/. Data are available upon request.

## References

[1] Burge C, Karlin S (1997). Prediction of complete gene structures in human genomic DNA. J Mol Biol.

[2] Burge CB, Karlin S (1998). Finding the genes in genomic DNA. Curr Opin Struct Biol.

[3] Parra G, Blanco E, Guigo R (2000). GeneID in Drosophila. Genome Res.

[4] Krogh A (1997). Two methods for improving performance of an HMM and their application for gene finding. Proc Int Conf Intell Syst Mol Biol.

[5] Lukashin AV, Borodovsky M (1998). GeneMark.hmm: New solutions for gene finding. Nucleic Acids Res.

[6] Majoros WH, Pertea M, Salzberg SL (2004). TigrScan and GlimmerHMM: two open source ab initio eukaryotic gene-finders. Bioinformatics.

[7] Salamov AA, Solovyev VV (2000). Ab initio Gene Finding in Drosophila Genomic DNA. Genome Res.

[8] Korf I (2004). Gene finding in novel genomes. BMC Bioinformatics.

[9] Stanke M (2003). Gene Prediction with a Hidden-Markov Model: Universitat Gottingen; 2003, this is the dissertation to obtain the doctoral degree of the Faculty of Mathematics and Natural Sciences.

[10] Birney E, Durbin R (1997). Dynamite: a flexible code generating language for dynamic programming methods used in sequence comparison. Proc Int Conf Intell Syst Mol Biol.

[11] Yeh RF, Lim LP, Burge CB (2001). Computational Inference of Homologous Gene Structures in the Human Genome. Genome Res.

[12] Taher L, Rinner O, Garg S, Sczyrba A, Brudno M, Batzoglou S (2003). AGenDA: homology-based gene prediction. Bioinformatics.

[13] Morgenstern B, Rinner O, Abdeddaim S, Haase D, Mayer KFX, Dress AWM (2002). Exon discovery by genomic sequence alignment. Bioinformatics.

[14] Korf I, Flicek P, Duan D, Brent MR (2001). Integrating genomic homology into gene structure prediction. Bioinformatics.

[15] Parra G, Agarwal P, Abril JF, Wiehe T, Fickett JW, Guigo R (2003). Comparative Gene Prediction in Human and Mouse. Genome Res.

[16] Meyer IM, Durbin R (2002). Comparative ab initio prediction of gene structures using pair HMMs. Bioinformatics.

[17] Bafna V, Huson D (2000). The conserved exon method for gene finding. Proc Int Conf Intell Syst Mol Biol.

[18] Alexandersson M, Cawley S, Pachter L (2003). SLAM: Cross-Species Gene Finding and Alignment with a Generalized Pair Hidden Markov Model. Genome Res.

[19] El Allali A, Rose JR. MGC: a metagenomic gene caller. BMC Bioinformatics. 2013; 14(9):S6. 10.1186/1471-2105-14-S9-S6. 10.1186/1471-2105-14-S9-S6 PMC3698006 23901840

[20] Hoff KJ, Tech M, Lingner T, Daniel R, Morgenstern B, Meinicke P. Gene prediction in metagenomic fragments: A large scale machine learning approach. BMC Bioinformatics. 2008; 9(1):217. 10.1186/1471-2105-9-217. 10.1186/1471-2105-9-217 PMC2409338 18442389

[21] Liu Y, Guo J, Hu G, Zhu H. Gene prediction in metagenomic fragments based on the SVM algorithm. BMC Bioinformatics. 2013; 14(5):S12. 10.1186/1471-2105-14-S5-S12. 10.1186/1471-2105-14-S5-S12 PMC3622649 23735199

[22] Stanke M, Waack S (2003). Gene prediction with a hidden Markov model and a new intron submodel. Bioinformatics.

[23] Stanke M, Morgenstern B (2005). AUGUSTUS: a web server for gene prediction in eukaryotes that allows user-defined constraints. Nucleic Acids Res.

[24] Bowman MJ, Pulman JA, Liu TL, Childs KL (2017). A modified GC-specific MAKER gene annotation method reveals improved and novel gene predictions of high and low GC content in Oryza sativa. BMC Bioinformatics.

[25] Jiang N, Ferguson AA, Slotkin RK, Lisch D (2011). Pack-Mutator-like transposable elements (Pack-MULEs) induce directional modification of genes through biased insertion and DNA acquisition. Proc Natl Acad Sci U S A.

[26] Rocha EPC (2004). Codon usage bias from tRNA’s point of view: Redundancy, specialization, and efficient decoding for translation optimization. Genome Res.

[27] Augustus server. http://augustus.gobics.de/datasets/. Accessed 08 Sept 2016.

[28] Stanke M, Diekhans M, Baertsch R, Haussler D (2008). Using native and syntenically mapped cDNA alignments to improve de novo gene finding. Bioinformatics.

[29] Augustus download. http://augustus.gobics.de/binaries/old/. Accessed 08 Sept 2016.

[30] Ouyang S, Zhu W, Hamilton J, Lin H, Campbell M, Childs K (2007). The TIGR Rice Genome Annotation Resource: improvements and new features. Nucleic Acids Res.

[31] Yuan Q, Ouyang S, Wang A, Zhu W, Maiti R, Lin H (2005). The Institute for Genomic Research Osa1 Rice Genome Annotation Database. Plant Physiol.

[32] Phytozome. The Plant Comparative Genomics portal of the Department of Energy’s Joint Genome Institute. https://phytozome.jgi.doe.gov/pz/portal.html. Accessed 05 Nov 2016.

